# Long Non-Coding RNAs in Diagnosis, Treatment, Prognosis, and Progression of Glioma: A State-of-the-Art Review

**DOI:** 10.3389/fonc.2021.712786

**Published:** 2021-07-12

**Authors:** Sara Momtazmanesh, Nima Rezaei

**Affiliations:** ^1^ School of Medicine, Tehran University of Medical Sciences, Tehran, Iran; ^2^ Network of Immunity in Infection, Malignancy and Autoimmunity (NIIMA), Universal Scientific Education and Research Network (USERN), Tehran, Iran; ^3^ Research Center for Immunodeficiencies, Pediatrics Center of Excellence, Children’s Medical Center, Tehran University of Medical Sciences, Tehran, Iran; ^4^ Department of Immunology, School of Medicine, Tehran University of Medical Sciences, Tehran, Iran

**Keywords:** biomarker, glioma, glioblasoma, long non coding RNA, micro RNA, prognosis, survival, treatment

## Abstract

Glioma is the most common malignant central nervous system tumor with significant mortality and morbidity. Despite considerable advances, the exact molecular pathways involved in tumor progression are not fully elucidated, and patients commonly face a poor prognosis. Long non-coding RNAs (lncRNAs) have recently drawn extra attention for their potential roles in different types of cancer as well as non-malignant diseases. More than 200 lncRNAs have been reported to be associated with glioma. We aimed to assess the roles of the most investigated lncRNAs in different stages of tumor progression and the mediating molecular pathways in addition to their clinical applications. lncRNAs are involved in different stages of tumor formation, invasion, and progression, including regulating the cell cycle, apoptosis, autophagy, epithelial-to-mesenchymal transition, tumor stemness, angiogenesis, the integrity of the blood-tumor-brain barrier, tumor metabolism, and immunological responses. The well-known oncogenic lncRNAs, which are upregulated in glioma, are *H19*, *HOTAIR*, *PVT1*, *UCA1*, *XIST*, *CRNDE*, *FOXD2-AS1*, *ANRIL*, *HOXA11-AS*, *TP73-AS1*, and *DANCR*. On the other hand, *MEG3*, *GAS5*, *CCASC2*, and *TUSC7* are tumor suppressor lncRNAs, which are downregulated. While most studies reported oncogenic effects for *MALAT1*, *TUG1*, and *NEAT1*, there are some controversies regarding these lncRNAs. Expression levels of lncRNAs can be associated with tumor grade, survival, treatment response (chemotherapy drugs or radiotherapy), and overall prognosis. Moreover, circulatory levels of lncRNAs, such as *MALAT1, H19, HOTAIR, NEAT1, TUG1, GAS5, LINK-A*, and *TUSC7*, can provide non-invasive diagnostic and prognostic tools. Modulation of expression of lncRNAs using antisense oligonucleotides can lead to novel therapeutics. Notably, a profound understanding of the underlying molecular pathways involved in the function of lncRNAs is required to develop novel therapeutic targets. More investigations with large sample sizes and increased focus on *in-vivo* models are required to expand our understanding of the potential roles and application of lncRNAs in glioma.

## Introduction

Glioma is the most common malignant central nervous system (CNS) tumor with significant mortality and morbidity (1). Glioblastoma is the most common and aggressive type of glioma with a median overall survival of less than two years (2). Notwithstanding substantial advances, the exact molecular pathways involved in tumorigenesis, tumor suppression, and treatment response are not fully elucidated in glioma, and patients commonly face a poor prognosis (3).

Non-coding ribonucleic acids (RNAs), comprising more than 97% of the human genome with various functions in physiological and pathological conditions, play a major role in glioma tumorigenesis (4). Non-coding RNAs are divided into the categories of short and long non-coding RNAs. The quintessential example of the former group are mi-RNAs, the role of which in glioma has been thoroughly investigated and reviewed (5, 6). In the past decade, long non-coding RNAs (lncRNAs) have drawn extra attention. More than 95% of the articles on lncRNAs and glioma retrieved from PubMed were published after 2017.

Lack of optimal treatment options in addition to specific and sensitive biomarkers (7) necessitates investigation of molecular pathways involved in glioma progression in the hope of finding novel therapeutic and diagnostic targets. LncRNAs may stand as prospective candidates for this purpose.

In this review, after providing a brief background on lncRNAs and their functions, we reviewed their role in various oncogenic processes. We also assessed their role in determining treatment response, survival, and prognosis. Lastly, the diagnostic and prognostic value of circulatory lncRNAs and potential therapeutic applications of modulation of lncRNAs expression *in-vivo* were investigated.

## An Overview on LncRNAs

LncRNAs are non-protein-coding RNAs with more than 200 nucleotides that are transcribed mainly by RNA polymerase II. As a result, lncRNAs, like messenger (m)RNAs, are typically polyadenylated and capped (8). However, compared to mRNAs, they are more nuclear-localized, more scarce, less evolutionary conserved, and contain fewer exons (9).

**Figure 1:**
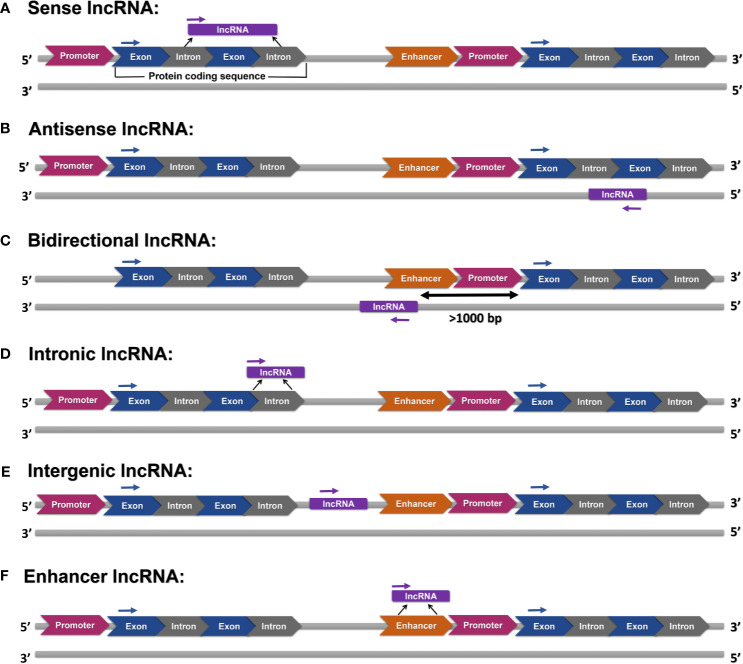
Various categories of lncRNAs: **(A)** sense lncRNAs are transcripts of one or more exons of protein-coding genes, **(B)** antisense lncRNAs are transcripts of the opposite strand of protein-coding or non-protein-coding genes, **(C)** bidirectional lncRNAs are transcribed in an opposite direction, and their transcription is initiated at more than 1000 base pairs (bp) far from the promoter region of a protein-coding gene, **(D)** intronic lncRNAs are transcribed from introns, **(E)** intergenic lncRNAs are transcribed from sequences without any overlap with annotated protein-coding genes, and **(F)** enhancer lncRNAs are produced from enhancer regions.

LncRNAs can be categorized into six groups according to their location on the genome, namely (a) sense, (b) antisense, (c) bidirectional (d) intronic, (e), and (f) enhancer lncRNAs (**Figure 1**).

LncRNAs have various functions in the nucleus and cytoplasm. In the nucleus, they play a role in chromatin remodeling, modulating chromosomal interactions, transcription regulation, and regulation of gene expression at a post-transcriptional level by altering the function and integrity of nuclear bodies. In the cytoplasm, they are involved in mRNA turnover, translation, and post-translational modification regulation. To regulate mRNA stability, competing endogenous RNAs (ceRNA) can modulate mi-RNA availability *via* vying with mRNAs for mi-RNA and act as mi-RNA sponges. Moreover, lncRNAs can recruit mRNA degradation-associated proteins or act as decoys for RNA binding proteins involved in mRNA decay machinery. lncRNAs can affect translation through interacting with ribosomes or modifying mRNAs to activate their translation. lncRNAs are also involved in a variety of post-transcriptional modifications, most importantly phosphorylation and ubiquitination (9, 10).

## LncRNAs in Glioma

### MALAT1

#### Overview - Expression Pattern

Metastasis-associated lung adenocarcinoma transcript 1 (*MALAT1*), also known as nuclear enriched abundant transcript (*NEAT*)2, is an intergenic lncRNA located on chromosome 11q13. Originally, *MALAT1* was introduced as a prognostic marker in non-small cell lung cancer. It is associated with several cancers such as breast, ovarian, prostate, pancreatic cancers, and leukemia (11).

Both higher (12, 13) and lower (14, 15) *MALAT1* expression are found in glioma than non-neoplastic tissue. Similarly, among glioma cell lines, glioma stem cell lines showed either lower (14) or higher (15) *MALAT1* expression than parental cells. Cancer stem cells showed upregulation of *MALAT1* compared to differentiated cancer cells in glioblastoma (16). Notably, between different glioblastoma cell lines, *MALAT1* expression was higher in U87 than U251 (15).

#### Role in Tumor Pathology

**Cell cycle and proliferation**: *MALAT1* knockdown resulted in tumor growth inhibition (17) and induced cell cycle arrest at G1/S phase in glioblastoma (U251) cells putatively *via* regulating the *miR-124*/zinc finger E−box binding homeobox 2 (ZEB2) axis (18). Nano complexes of si-*MALAT* induced G2/M, in addition to G1, cell cycle arrest (16). Accordingly, *MALAT1* expression enhanced tumor proliferation by upregulating Rap1b and zinc-fingers and homeoboxes 1 (ZHX1) by sponging miR-101 in glioma (19) and *miR-199a* in glioblastoma (20), respectively. Notably, ZHX1 plays a key role in glioblastoma progression (21).

In contrast to the above-mentioned mechanisms, Han et al. reported that knockdown of *MALAT1* induced tumor proliferation in U87 and U252 cell lines, potentially by suppressing the extracellular signal-regulated kinases (ERK)/mitogen-activated protein kinase (MAPK) signaling pathway (22). In line with their finding, Cao et al. found that *MALAT1* can have tumor-suppressing effects by reducing *miR-155* expression and increasing expression of FBXW7 tumor suppressor, which interacts with several molecules involved in cellular growth, development, stemness, and cell cycle (14, 23, 24).

**Apoptosis**: *MALAT1* knockdown increased apoptosis and expression of apoptotic regulators, including MYC and CCND1 (encoding cyclin D1) in glioma (13). Inhibition of apoptosis by *MALAT1* can also be regulated *via* the *MALAT1*/*miR-101*/Rap1B axis (19) and the miR-124/ZEB2 axis (18). Moreover, inhibition of *MALAT1* by si-*MALAT1* resulted in a significant decrease in the levels of several molecules involved in apoptosis, such as Bcl-2, inhibitors of apoptosis proteins family, and heat shock protein (HSP) 70 (16). Additionally, *MALAT-1* knockdown resulted in lower expression of Bax and higher expression of Bcl-2 *via* regulating the *miR-199a*/ZHX1 axis (20).

**Autophagy**: Although autophagy may induce cytotoxic effects, it has also been suggested to promote the progression and viability of glioma in stressful environments (25). *MALAT1* is found to promote tumor progression by enhancing autophagy. Sponging miR-101 not only enhanced tumor proliferation by upregulating Rap1b (19), but also induced higher expression of autophagy-associated genes (Stathmin 1, RAB5A, and ATG4D) (26). *MALAT1* acts as a sponge for miR-384 as well. Inhibition of miR-384 activity induced autophagy by putatively interfering with Golgi membrane protein 1 (GOLM1) and led to increased migration and invasion of glioma cells (27, 28).

**Invasion and metastasis**: Knockdown of *MALAT1* suppressed migration and invasion of glioma cells *via* several mechanisms, such as inhibiting autophagy *via* regulating the *miR-384*/GOLM1 axis (27). *MALAT1* played a critical role in tumor migration. Notably, Wnt inhibitory factor 1 (WIF1) regulated *MALAT1* expression through the non-canonical Wnt signaling pathway (29). *MALAT1* also promoted tumor invasiveness *via* regulating the *miR-199a*/ZHX1 axis (20).

Conversely, Han et al. found that *MALAT1* knockdown increased invasion and proliferation of glioma cells in addition to inducing higher expression of matrix metalloproteinase (MMP)2 (22).

**Stemness**: *MALAT1* overexpression promoted proliferation of glioma stem cells (30) by enhancing SRY-related HMG-box (SOX)-2 expression *via* inhibiting tumor suppressor *miR-129*, which led to increased tumor proliferation and viability (17). In addition to SOX-2, *MALAT1* downregulation has shown inhibitory effects on the expression of Nestin (another stemness marker) and proliferation of glioma stem cell lines by activating the ERK/MAPK signaling pathway, which is a key pathway in tumor development (15, 31).

**Blood tumor barrier (BTB)**: *MALAT1* knockdown led to enhanced BTB permeability and reduced expression of tight junction proteins in glioma endothelial cells *via* upregulating *miR-140*. The effect of *miR-140* on BTB is mediated by inhibiting expression of nuclear factor YA (NFYA), a regulator of BTB integrity, resulting in increased expression of tight junction proteins (12).

**Immunology**: In microglia, deactivating *MALAT1* using si-*MALAT1* modulated the *miR-129-5p*/high mobility group box 1 protein (HMGB1) axis resulting in a reduced inflammatory response (30).

#### Clinical Applications

**Circulatory biomarker**: Compared to healthy controls, glioma patients had lower serum *MALAT1* levels (14). Serum levels of *MALAT1* have also been used as diagnostic and prognostic biomarkers in some other cancers (32–34).

**Prognostic value**: Meta-analyses showed that increased *MALAT1* expression could predict poor overall survival (35, 36) and higher “tumor, node, metastasis” (TNM) stage (37) in glioma patients. Moreover, tissue expression levels of *MALAT1* positively correlated with tumor grade according to the world health organization (WHO) classification and tumor size (18, 38). Serum levels of *MALAT1* were also positively associated with WHO grade, tumor size, functional impairment (14), and overall and recurrence-free survival (39). However, Shen et al. did not find a significant association between serum levels of *MALAT1* and 2-year survival or disease-free survival (40).

**Determining treatment response**: *MALAT1* plays a major role in tumor chemosensitivity with a higher expression in temozolomide (TMZ)-resistant glioblastoma cells. Its knockdown reduced TMZ resistance both *in vivo* and *in vitro* (16, 41). Additionally, elevated serum levels of *MALAT1* predicted chemoresistance (39). *MALAT1* can modulate treatment response *via* several mechanisms. It can inhibit the *miR-101* pathway through direct binding, resulting in increased apoptosis and suppressed cell growth (41), downregulate *miR-203* leading to upregulation of thymidylate synthase (39), and regulate ZEB1 expression (42). Furthermore, inhibition of *MALAT1* resulted in decreased expression of multi-drug resistance (MDR)-associated protein 1 (MRP1), a drug efflux pump associated with TMZ resistance (16).

***In-vivo* therapeutic applications**: Silencing *MALAT1* suppressed proliferation and malignant behavior of glioma, leading to decreased tumor volume and increased survival (18) *in vivo via* regulating several pathways, including miR-199a/ZHX1 (20), *miR-129*/SOX2 (17), and *miR-384*/GOLM1 (27). In tumor xenograft models, nano complexes of si-*MALAT1* targeting cancer stem cells TMZ sensitivity and survival in addition to proliferation inhibition (16, 41), while *MALAT1* overexpression induced TMZ resistance (43).

### H19

#### Overview - Expression Pattern

H19 is an imprinted intergenic lncRNA located on chromosome 11p15.5, which is generally expressed by the maternal allele. H19 has well-known oncogenic effects in several cancers, such as hepatocellular carcinoma, bladder, breast, gastric, and colorectal cancers (44).

The expression of *H19* was higher in glioma tissue (low and high grade) (45, 46) and cell lines, including U251 and U87MG cells (47, 48).

#### Role in Tumor Pathology

**Cell cycle and proliferation**: Knockdown of *H19* inhibited glioma cell growth, indicating that *H19* interacts with the cell cycle and enhances glioma proliferation (45–47). H19 downregulation induced G0/G1 cell cycle arrest putatively *via* inhibiting the WNT/β-catenin signaling pathway (48). The oncogenic effects of *H19* can be mediated *via* increased expression of *miR-675*, which regulates expression of cadherin 13 (45, 49) and vitamin D receptor (a transcriptional factor involved in several cell signaling pathways) (50). *H19* also affects tumor proliferation through downregulating *miR-152* (51) and upregulating tumor promoter inhibitor of apoptosis-stimulating protein of p53 (iASPP) *via* targeting *miR-140* (52).

**Apoptosis**: Downregulation of *H19* induced apoptosis and stopped the cell cycle (52) mainly by suppressing the Wnt/β-catenin signaling pathway (48), in addition to iASPP upregulation (52). Knockdown of *H19 via* siRNA resulted in increased TMZ-induced apoptosis rate in U87MG and U251 cell lines in glioblastoma (47).

**Autophagy**: *H19* overexpression suppressed autophagy of glioma cells *via* regulating the mammalian target of rapamycin (mTOR)/Unc-51 like autophagy activating kinase 1 (ULK1) axis by inducing increased ULK1 phosphorylation and inhibiting mTOR phosphorylation (53).

**Invasion and metastasis**: *in-vitro* Matrigel invasion assay showed that overexpression of H19 enhanced the invasiveness of glioblastoma cells (46). Knockdown of *H19* inhibited glioma metastasis *in vivo* and *in vitro* (52). *H19* downregulation inhibited the Wnt/β-catenin signaling pathway (48). Additionally, *H19* diminished the inhibitory effect of *miR-181d* on β-catenin by sponging this tumor suppressor miRNA (54). *H19* also upregulated *miR-675* (49) and downregulated tumor suppressor *miR-152* (51).

**Epithelial-mesenchymal transition (EMT) process**: EMT, a major role player in tumorigenesis by promoting metastasis, tumor stemness, and chemoresistance, is characterized by increased expression of epithelial markers, such as E-cadherin, and decreased mesenchymal markers, such as N-cadherin, vimentin, and ZEB1/2 (55).

*H19* overexpression enhanced mesenchymal markers, namely N-cadherin and vimentin, expression. The potential underlying mechanism was sponging *miR-130a-3p*, which increased the expression of SOX4 (56), a critical transcription factor in the EMT process (57). Additionally, *H19* silencing suppressed EMT (increased E-cadherin expression and decreased ZEB1 and vimentin expression) through inhibiting the Wnt/β-catenin pathway activity (58).

**Stemness**: *H19* was highly expressed in glioblastoma stem cells (CD133+ cells) and promoted stemness (46). Accordingly, its knockdown led to decreased expression of stemness markers, including CD133, NANOG, Oct4, and SOX2 (47).

**Angiogenesis**: *H19* plays a key role in angiogenesis in glioma *via* several mechanisms, including inhibiting *miR-29a* and *miR-138*. The former upregulated vasohibin-2 (VASH2) (an angiogenic factor) (59), and the latter induced higher expression of hypoxia-inducible factor (HIF)-1α and vascular endothelial growth factor (VEGF) (60). Furthermore, in glioblastoma, *H19* reduced expression of Nkd1, which is a Wnt pathway inhibitor, *via* EZH2-mediated epigenetic regulations (61), and its overexpression increased angiogenesis in *in-vitro* investigations (46).

#### Clinical applications

**Circulatory biomarker**: While circulatory H19 levels were a reliable prognostic indicator, to the best of our knowledge, their diagnostic value has not been investigated in glioma (40, 62). Moreover, *H19* plasma levels are proposed as a diagnostic biomarker for gastric cancer (63).

**Prognostic value**: *H19* overexpression in glioma tissue was associated with poor overall and progression-free survival and more advanced tumor stage (45, 46, 48, 64). Moreover, serum levels of *H19* showed a significant positive correlation with tumor grade (62). However, Shen et al. did not find a significant association between serum levels of H19 and 2-year or disease-free survival (40).

**Determining treatment response**: TMZ-resistant glioma cell lines had higher *H19* expression (58, 65, 66). *H19* induced chemoresistance by promoting EMT through the Wnt/B-catenin pathway (58). *H19* silencing reduced chemoresistance and increased TMZ-induced apoptosis through inhibiting the NF-κB signaling pathway (47, 65) and downregulating chemoresistance-associated genes (*MDR*, *MRP*), and ATP-binding cassette subfamily G member 2 *(ABCG2*) (66).

***In-vivo* therapeutic applications**: H19 promoted proliferation, migration, and angiogenesis in tumor xenograft investigations, while its knockdown inhibited tumor progression (46, 51, 52). Modulating the *miR-342*/Wnt5a/β-catenin axis is one of the proposed mechanisms for the oncogenic effect of H19 on tumor growth, metastasis, and angiogenesis *in vivo* (67). Notably, in a study assessing the therapeutic effects of phenformin in glioblastoma, phenformin was found to inhibit tumor stemness through downregulating H19 and high mobility group A (HMGA)2 (68).

### MEG3

#### Overview - Expression Pattern

Maternally expressed gene 3 *(MEG3)*, also known as gene-trap locus 2 (GTL2) in mice, is a maternally imprinted intergenic lncRNA (like *H19*) located on chromosome 14q32.3. *MEG3* has shown anti-tumoral effects in several cancers, such as lung, breast, liver, gastric, colorectal, ovarian, and cervical, in addition to glioma (69).

*MEG3* is downregulated in glioma tissue and cell lines (70–74). Its downregulation can be a result of hypermethylation (75).

#### Role in Tumor Pathology

**Cell cycle and proliferation**: *MEG3* plays a substantial role in glioma proliferation and cell cycle regulation. Deletion of *MEG3* increased tumor cell growth and enhanced cell proliferation in normal human astrocytes (76). *MEG3* overexpression led to cell cycle arrest in the G2/M phase in U251 cells (77) and inhibited cell proliferation of glioma cells (71).

*MEG3* upregulated key tumor suppressors mainly by interacting with the regulatory miRNAs (69). The p53 protein, encoded by the tumor suppressor protein p53 (*TP53*) gene, is involved in several cellular protective mechanisms, including inducing cell cycle arrest, DNA repair, and apoptosis (78). MEG3 is required for the activation of the p53 pathway (73). Decreased *MEG3* expression due to DNA (cytosine-5)-methyltransferase 1 (DNMT1)- mediated hypermethylation inhibited the p53 pathway in glioma (75). Correspondingly, *MEG3* overexpression increased TP53 mRNA levels and suppressed cell proliferation in U251 and U87 cell lines (73).

*MEG3* is also associated with phosphatase and TENsin homolog (PTEN) expression, negatively regulating the phosphoinositide 3-kinase (PI3K). *miR-19a* is found to have repressive effects on PTEN expression. *MEG3* acted as a ceRNA for *miR-19a*, recovering its inhibitory effects on PTEN expression. It resulted in decreased cell proliferation, cell cycle arrest at the G1/S phase, and increased apoptosis (79). Moreover, the regulatory role of the *miR-377*/PTEN axis was identified in U251 cells (77). *MEG3* overexpression also upregulated metastasis suppressor 1 (MTSS1) by downregulating *miR-96-5p* (71).

Furthermore, EZH2-mediated H3K27me3 enrichment (tri-methylation of lysine 27 on histone H3 protein) of the *MEG3* gene downregulated this lncRNA. *MEG3* inhibited *miR-21-3p* expression resulting in reduced tumor proliferation and invasion (70).

*MEG3* also modulated Wnt/β-catenin signaling, leading to enhanced tumor proliferation following *MEG3* downregulation in glioma (76). *MEG3* also increased the expression of SMARCB, which suppressed tumor proliferation and migration by sponging miR-6088 (80).

**Apoptosis**: *MEG3* overexpression induced apoptosis in glioma cell lines, mainly regulated by the interaction of *MEG3* and p53 activation (72, 73). Apoptosis was inhibited after silencing of *MEG3* in U118 cells. At the same time, it was enhanced following *MEG3* overexpression in U251 cells through induction of cell cycle arrest at G2/M phase and increasing mRNA levels of caspase 8/3 and TP53, both playing a crucial role in cell apoptosis (73, 77).

**Autophagy**: *MEG3* overexpression promoted autophagy and induced higher expression of autophagy-associated proteins, including ATG3, ATG5, Beclin-1, LAMP1, and LC3 (72, 81). 

**Invasion and metastasis**: Silencing of *MEG3* increased migration and invasion in glioma (77). The interaction of *MEG3* and tumor suppressors plays a key role in tumor invasion and metastasis. *MEG3* upregulation inhibited metastasis *via* regulating the *miR−96−5p*/MTSS1 axis (71). Its downregulation promoted migration and invasion *via* modulating the *miR-19a*/PTEN axis through acting as a ceRNA for *miR-19a* (79). Downregulating *miR-21-3p* (70) and enhanced expression of SMARCB due to sponging *miR-6088* (80) are among other proposed mechanisms by which *MEG3* blocks tumor invasion and migration. Nevertheless, since *MEG3* overexpression induced autophagy (72), it increased migration and invasion in U87 and U251 cells *via* this mechanism (81).

**EMT**: *MEG3* overexpression led to reduced EMT with decreased expression of N-cadherin, vimentin, Snail-1, and -catenin (only reported in U251 cells) and increased expression of E-cadherin in U87 and U251 cells (77, 80). Accordingly, *MEG3* silencing promoted EMT *via* regulating the miR-377/PTEN axis (77) in addition to inducing autophagy (81). However, in the U118 cell line, MEG3 overexpression did not significantly change the EMT markers (77). Conversely, Yang et al. reported that *MEG3* overexpression induced a more mesenchymal cell-like morphology and increased expression of ZEB1/2. Notably, inhibition of autophagy suppressed *MEG3*-induced EMT (81).

#### Clinical Applications

**Circulatory biomarker**: To the best of our knowledge, the biomarker value of circulatory *MEG3* has not been investigated in glioma. Although, circulatory *MEG3* has shown biomarker value in other cancers, such as colorectal (82), gastric (83), breast (84), bladder (34), and pancreatic (85).

**Prognostic value:** Lower *MEG3* expression was associated with higher WHO grade, older age at the time of diagnosis, low Karnofsky performance score (KPS), isocitrate dehydrogenase (IDH) wild-type, tumor recurrence, and poor overall survival (72, 74, 76, 86).

**Determining treatment response**: *MEG3* also determined chemoresponse in glioma. TMZ-resistant glioblastoma had a lower *MEG3* expression compared to TMZ responders (39). Moreover, enhanced *MEG3* expression increased chemosensitivity to cisplatin while *MEG3* silencing *via* si-RNA induced chemoresistance (87).

***In-vivo* therapeutic applications**: Targeting epigenetic regulation of *MEG3* expression can provide novel therapeutic choices for glioma. For example, the DNA methylation inhibitor 5-Aza-2’-deoxycytidine (5-AzadC) reduced the abnormal *MEG3* promoter hypermethylation and prevented low *MEG3* expression (75). Moreover, administration of synthetic miRNAs, such as miR-377 mimic, can help increase MEG3 expression and inhibit tumor migration and invasion (77). Notably, the anti-tumoral effect of tunicamycin was mediated through MEG3 upregulation (88).

### HOTAIR

#### Overview - Expression Pattern

HOX transcript antisense intergenic RNA (*HOTAIR*), an oncogenic lncRNA located on chromosome 12q13.13, is the first identified trans-acting lncRNA with widely explored roles in breast, lung, cervical, colorectal, and bladder cancers, and glioma (89).

Glioma tissue (both low-grade and high-grade), as well as glioma cell lines (U867 and U251), had higher *HOTAIR* expression compared to non-neoplastic brain tissue (90–94). Investigating several datasets showed that DNA methylation, particularly methylation of CpG islands, regulated *HOTAIR* expression with demethylation resulting in increased transcription. Moreover, *HOXA9*, an oncogenic regulator in glioma (95), also induced *HOTAIR* expression *via* interacting with its promoter (91).

#### Role in Tumor Pathology

**Cell cycle and proliferation**: *HOTAIR* is required for the formation of glioblastoma (93) and influenced the cell cycle (96) by regulating molecules having a role in its different phases (97). Several mechanisms have been suggested for the involvement of *HOTAIR* in the cell cycle. *HOTAIR* can promote cell growth by suppressing EZH2 [the catalytic component of polycomb repressive complex 2 (PRC2)] activity, which leads to chromatin condensation by binding to the PRC2 complex (98). *HOTAIR* is found to have reciprocal interactions with *miR-15-b* and p53. *miR-15-b* positively regulated p53. Both of these molecules inhibit tumor proliferation and invasion, while *HOTAIR* activity can suppress their impact (99). Moreover, *HOTAIR* suppressed the β-catenin pathway, leading to cell cycle arrest and repression of invasion, putatively by downregulating Nemo-like kinase (NLK) in glioblastoma (93). *HOTAIR* silencing also decreased cyclin D1 expression by upregulating *miR-219* (100). Additionally, *HOTAIR* promoted tumor proliferation by acting as a ceRNA for *miR-218*, resulting in upregulation of PDE7A (101). *HOTAIR* also activated the mTOR pathway *via* regulating *miR-125a*, resulting in increased tumor viability (102). *HOTAIR* also downregulated tumor suppressor programmed cell death 4 (PDCD4), leading to increased growth and proliferation of glioma stem cells (103).

**Apoptosis**: *HOTAIR* silencing induced apoptosis with several mechanisms. Upregulating PDE7A *via* decreasing *miR-218* expression (101), enhancing *miR-219* and Bax expression (100), regulating the *miR-15-b*/p53 axis (99), and activating the mTOR pathway (102) are among the possible underlying mechanisms.

**Angiogenesis**: *HOTAIR* induced angiogenesis *via* increasing expression of VEGFA in glioma, which was suppressed after *HOTAIR* silencing (104). Correspondingly, downregulation of *HOTAIR* inhibited the angiogenesis ability of human umbilical vein endothelial cells putatively by sponging miR-126-5p (105).

**Invasion and metastasis**: *HOTAIR* downregulation inhibited tumor invasiveness and migratory abilities. Several molecular mechanisms have been suggested for the positive effect of *HOTAIR* on tumor progression. Downregulating NLK, resulting in increased activation of the β-catenin pathway (93), suppressing *miR-125a*, leading to increased activity of the mTOR pathway (102), and inhibiting the tumor suppressor *miR-15b*/p53 axis (99) are among these mechanisms. Moreover, overexpression of *HOTAIR* was also associated with higher levels of MMP-7 and MMP-9 (106). Regulating the *miR-218*/PDE7A axis (101) and glutamine metabolism *via* downregulating *miR-126-5p*, which resulted in glutaminase upregulation (105), in addition to inhibiting tumor suppressor PDCD4 (103), are other proposed mechanisms.

**BTB**: *HOTAIR* knockdown decreased expression of tight junction proteins, including ZO-1, occludin, and claudin-5, and led to a discontinuous distribution pattern among them by negatively regulating *miR-148b-3p* and upregulating upstream stimulatory factor (USF)1 (107).

**Metabolism**: *HOTAIR* regulated glutamine metabolism, which is essential for glioma progression, by sponging miR-126-5p (105).

#### Clinical Applications

**Circulatory biomarker**: Serum *HOTAIR* levels were significantly higher in glioblastoma patients than controls and correlated with *HOTAIR* expression within the glioblastoma tissue and glioma grade (108). Circulatory *HOTAIR* has also been proposed as a potential biomarker for other cancers (109–111).

**Prognostic value**: In addition to the higher circulatory *HOTAIR* levels in higher glioma grades (108), several investigations, including those with large datasets, found that *HOTAIR* is far more expressed in high-grade than low-grade glioma tissue (91, 93, 96, 106). Moreover, IDH-wild type cases, which typically have a poor prognosis, had higher expression levels of *HOTAIR* (91). Higher *HOTAIR* expression was an independent predictor of reduced overall survival in glioblastoma (91). Additionally, two single-nucleotide polymorphisms (SNP) of *HOTAIR* (rs920778 CT and rs12826786 CT genotypes) were also associated with more prolonged overall survival in patients with WHO grade III anaplastic oligodendroglioma (112).

**Determining treatment response**: Expression of *HOTAIR* was higher in non-TMZ responder glioblastoma patients compared to responders (39). *HOTAIR* downregulation induced increased chemosensitivity to TMZ treatment, the underlying mechanism of which may be *HOTAIR* acting as ceRNA for miR-126-5p (105). Moreover, *HOTAIR* was found to induce higher expression of HK2 *via* downregulating *miR-125*. Increased hexokinase 2 (HK2) expression is associated with chemoresistance putatively through HK-2 mediated lactate production and mitochondria permeability transition pore opening (113). Notably, in addition to *HOTAIR*, the expression of HK-2 is also related to other lncRNAs, including *MALAT1*, *UCA1*, and *PVT1*.

***In-vivo* therapeutic applications**: *In vivo*, knockdown of *HOTAIR* using shRNA inhibited tumor growth and invasiveness and enhanced chemosensitivity (93, 113). Notably, promoter demethylation using 5-Aza-2’-deoxycytidine, which is typically used in the treatment of leukemia, affected the expression of *HOTAIR* (91). The decreased *HOTAIR* expression was associated with inhibition of invasiveness, angiogenesis, and chemoresistance (105). Of note, *HOTAIR* downregulation can mediate the tumor-suppressive effects of some miRNAs, such as *miR-326* (90).

### PVT1

#### Overview - Expression Pattern

Plasmacytoma variant translocation 1 (*PVT1*) is an intergenic lncRNA located on chromosome 8q24, a well-known cancer-associated region. The role of *PVT1* has been explored in several cancers such as leukemia, colon, hepatocellular, breast, lung, and ovarian cancers (114).

Several studies, including investigations of large datasets (115), found higher *PVT1* expression in glioma tissue and cell lines than normal (116–120).

#### Role in Tumor Pathology

**Cell cycle and proliferation**: *PVT1* downregulation inhibited tumor growth and expansion both *in vitro* and *in vivo* (117) and induced cell cycle arrest at the G1 phase (117–119, 121). The interactions of *PVT1* with some miRNAs can mediate its positive effect on tumor proliferation. For instance, *PVT1* downregulated *miR-128-1-5p* leading to increased polypyrimidine tract-binding protein 1 (PTBP1) expression (117). *PVT1* silencing also modulated the *miR-128-3p*/Gremlin 1 (GREM1) axis resulting in inhibition of the bone morphogenetic protein (BMP) signaling pathway and tumor growth (121). Furthermore, *PVT1* negatively regulated *miR−200a*, which has a critical role in glioma development (118). *PVT1* also upregulated *miR-190a-5p* and *miR-488-3p*, resulting in inhibited expression of myocyte enhancer factor 2C (MEF2C), an oncogenic factor in glioma (119).

**Apoptosis**: Downregulation of *PVT1* promoted apoptosis and DNA damage *via* increasing expression of Bax and cleaved caspase-3 protein and decreasing Bcl-2 expression. The stimulatory effect of *PVT1* knockdown on apoptosis can be mediated *via* several pathways, including regulating the *miR-128-1-5p*/PTBP1 axis (117) and the expression of *miR-128-3p* (121), *miR-190a-5p*, and *miR-488-3p* (119).

**Autophagy**: *PVT1* overexpression increased expression of autophagy-associated proteins, namely Atg7 and Beclin1, by inhibiting *miR-187* in glioma vascular endothelial cells (122).

**Invasion and metastasis**: *PVT1* induced tumor invasiveness *via* modulating several target molecules and signaling pathways (118). *PVT1* silencing reduced tumor migration and invasiveness *via* sponging *miR-128-3p*, which inhibited GREM1 and inhibition of the BMP signaling pathway (121). Moreover, *PVT1* silencing suppressed invasion, migration, and expression of MMP-2 and MMP-9 *via* upregulating *miR-128-1-5p*, which restrained expression of PTBP1 (117). In another proposed regulatory network, *PVT1* knockdown reduced tumor invasiveness and migration *via* upregulating tumor suppressor *miR-424* (120). *PVT1* knockdown upregulated *miR-190a-5p* and *miR-488-3p*, resulting in inhibited expression of MEF2C. MEF2C upregulates promoter activity of JAGGED 1, which is involved in tumor malignant behavior (119). *PVT1* upregulation accelerated migratory abilities of glioma *via* downregulating up-frameshift protein1 (UPF1), which is a key role player in the nonsense-mediated mRNA decay (NMD) (123).

**Angiogenesis**: Glioma vascular endothelial cells had a higher *PVT1* expression. *PVT1* overexpression promoted angiogenesis *via* degrading *miR-186*, resulting in upregulated Atg7 and Beclin1 expression (122). Moreover, PVT1 overexpression led to upregulation of connective tissue growth factor (CTGF) and angiopoietin 2 *via* targeting *miR-26b* (124).

#### Clinical Applications

**Circulatory biomarker**: To the best of our knowledge, the biomarker value of circulatory *PVT1* has not been investigated in glioma. Nevertheless, circulatory *PVT1* levels had diagnostic and prognostic value in some cancers (125, 126).

**Prognostic value**: Higher expression of *PVT1* was an indicator of poor prognosis (116) and survival (127) in glioma. Patients with higher glioma grade, metastasis, or IDH wild type glioma had higher tissue expression of *PVT1* (116, 119, 121, 123, 128). Higher *PVT1* expression positively correlated with Ki-67 level and the number of *TP53* mutations (127). However, *PVT1* expression was not associated with gender, age, KPS score, or tumor size (116). Only a few studies have evaluated the prognostic role of *PVT1* SNPs in glioma. Ding et al. reported that while rs13255292 and rs4410871 increased susceptibility to glioma in the Chinese Han population, they do not have a prognostic value (129).

**Determining treatment response**: *In vitro*, SHG-44 cells resistant to paclitaxel had higher *PVT1* expression, and *PVT1* knockdown enhanced chemoresponse (130).

***In-vivo* therapeutic applications**: *In vivo*, silencing of *PVT1* in nude mice with tumor xenograft resulted in decreased tumor volume and weight, which may be mediated *via* the interaction of *PVT1* with *miR-128-1-5p* (117), *miR-128-1-3p* (121), and *miR-424* (120). Additionally, silencing of *PVT1* in addition to *miR-190a-5p* and *miR-488-3p* mimics prolonged survival and reduced tumor volume in mice with tumor xenograft (119).

### UCA1

#### Overview - Expression Pattern

Urothelial carcinoma associated 1 (*UCA1*), located on chromosome 19p13.12, is an intergenic lncRNA involved in several cancers, such as lung, breast, gastric, and colorectal cancers, as well as glioma (131).

Upregulation of *UCA1* is reported in glioma tissue and cell lines compared with the normal brain samples (132–136).

#### Role in Tumor Pathology

**Cell cycle and proliferation**: *UCA1* interacted with the cell cycle. Its knockdown inhibited tumor proliferation, and its overexpression had the opposite effect both *in vitro* and *in vivo* (135, 136). *UCA1* knockdown induced G0/G1 cell cycle arrest and downregulation of cyclin D1 (132). Cyclin D1 is also involved in the Wnt/β-catenin signaling, which is inhibited following *UCA1* knockdown (135). Additionally, sponging tumor suppressor *miR-122* (133), *miR-135a* (136), and enhancing iASSP expression *via* downregulating *miR-182* (137) also contribute to the underlying mechanism of the positive effect of *UCA1* expression on tumor proliferation.

**Apoptosis**: Silencing *UCA1* facilitated apoptosis and reduced cell viability, and its overexpression had the opposite effect (135, 138). *UCA1* enhanced CDK6 expression *via* sponging *miR‐193a*. Notably, CDK6 triggers PI3K/AKT, MAPK, and Notch signaling pathways (138).

**Invasion and metastasis**: *UCA1* overexpression increased invasion and migration (139), while its silencing inhibited tumor progression *via* several mechanisms (134, 136, 138). *UCA1* acted as an endogenous sponge for several tumor suppressor miRNAs, such as *miR-122*, *miR-204-5p*, and miR-135a. Therefore, *UCA1* suppressed the inhibitory effect of *miR-204-5p*, *miR-135a*, and *miR-2016* on ZEB1, HOXD9, and CLOCK, respectively, which resulted in their upregulation (133, 134, 136, 139). Moreover, some of the key signaling pathways for tumor progression, including the Wnt/β-catenin, PI3K/AKT, MAPK, and notch signaling pathways, were suppressed following *UCA1* knockdown (135). Regulation of the *miR-193/*CDK6 axis mediated the positive effect of *UCA1* on the three latter signaling pathways (138). Additionally, *UCA1* enhanced the expression of tumor inducer iASSP expression *via* inhibiting *miR-182* expression (137).

**EMT**: *UCA1* knockdown inhibited the EMT process by increasing the expression of epithelial markers, i.e., E-cadherin, and decreasing the expression of mesenchymal markers, i.e., Slug, N‐cadherin, and vimentin (136, 139, 140). *UCA1* upregulated Slug *via* acting as a ceRNA for *miR-1* and *miR-203* (140). Moreover, *UCA1* upregulated EMT inducers, namely HOXD9, CLOCK, and ZEB1, *via* sponging *miR-135a* (136), *miR-206* (134), and *miR-204-5p*, respectively (139). Furthermore, *UCA1* is proposed to mediate the positive effect of TGF- β on EMT (140).

**Stemness**: Knockdown of *UCA1* reduced expression of the stemness markers due to regulating the *miR-1* and *miR-203*/Slug axis (140)

**Metabolism**: *UCA1* may play a major role in glycolysis, a well-known characteristic of glioblastoma, *via* modulating the *miR-182*/6-phosphofructo-2-kinase/fructose-2,6-biphosphatase 2 (PFKFB2) axis (141). Notably, the inhibition of glycolysis resulted in tumor-suppressive effects in glioma (142).

#### Clinical Applications

**Circulatory biomarker**: Diagnostic values of circulatory *UCA1* has been reported in other cancers, such as bladder, gastric cancer, and colorectal cancer (143–145). However, we did not find any reports in patients with glioma.

**Prognostic value**: Higher expression of *UCA1* was associated with higher glioma grade, poor prognosis, and survival (133, 135–137). However, it did not correlate with age, gender, tumor size, and KPS score (132).

**Determining treatment response**: *UCA1* overexpression-induced chemoresistance (shown by an increased IC50) to cisplatin and TMZ in U87 and SHG139 cells. This effect was attenuated by inhibiting the Wnt/β-catenin signaling pathway. Notably, TMZ sensitivity increased after *UCA1* knockdown (135).

***In-vivo* therapeutic applications**: In addition to the *in vitro* tumor suppressor effects of si-*UCA1* (132, 133), several studies showed that *UCA1* knockdown suppressed tumor progression and reduced tumor volume and weight in tumor xenograft models while its overexpression promoted tumor growth (134–136, 139). *miR135a*/HOXD9 (136), *miR-206*/CLOCK (134), and *miR-204-5p*/ZEB1 *(* 139) axes have been observed *in vivo* as well.

### Other lncRNAs

More than two hundred lncRNAs have been identified to be associated with glioma (146). Providing a detailed review of all of them would be beyond the scope of this review. Therefore, in this section, we give an overview of the other most investigated lncRNAs in glioma.

#### Role in Tumor Pathology

The roles of the most investigated oncogene lncRNAs and the so far discovered intermediate molecular pathways, in addition to their clinical applications, are summarized in **Table 1**. Overall, almost all oncogene lncRNAs regulate the cell cycle, promote tumor proliferation, inhibit apoptosis, and induce tumor invasiveness and migration. While lncRNAs such as *XIST* (162–172), *CRDNE* (173–179), *FOXD2-AS1* (186–194), *ANRIL* (195–197), *HOXA11-AS* (198–203), *TP73-AS1* (204–206), and *DANCR* (207–210) are only known as tumor inducers, the ultimate function of some lncRNAs is controversial. For instance, for *MALAT1*, *NEAT1*, and *TUG1*, both oncogenic and tumor suppressor effects have been reported.

**Table 1:** The most investigated oncogene lncRNAs and the so far discovered intermediate molecular pathways, in addition to their clinical applications.

lncRNA	Expression	Mechanism involved	Intermediate targets or signaling pathways	Clinical applications			References
				Circulatory biomarker	Treatment response	Prognostic
**MALAT1**	↑↓	Cell cycle and proliferation	miR-124/ZEB2 miR199/ZHX1 miR101/Rap1b miR199a miR-155/FBXW7 (tumor suppressor) ERK/MAPK signaling pathway (tumor suppressor)	Yes	Yes	Yes	(12–20, 22, 26, 27, 29, 30, 35, 36, 38, 39, 41)
		Apoptosis	miR-101/Rap1b miR-124/ZEB2 miR-199a/ZHX1/Bax, Bcl-2 expression of MYC, CCND1, Bcl-2, HSP- 70				
		Autophagy	miR-101/STMN1, RAB5A and ATG4D miR-384/GOLM1				
		Invasion and metastasis	miR-384/GOLM1 Wnt/calcium pathway miR-199a/ZHX1 Expression of MMP2 (tumor suppressor)				
		Stemness	miR-129/SOX-2 ERK/MAPK signaling pathway Expression of Nestin				
		Blood-tumor barrier	miR-140/NFYA/ZO-1, occludin and claudin-5				
		Immunology	miR-129-5p/HMGB1 (inducing inflammation)				
**H19**	↑	Cell cycle and proliferation	miR-342/Wnt5a/β-catenin (overall, H19 modulates the Wnt/β-catenin signaling pathway) miR-675/Cadherin13 miR-675/VDR miR-152 miR140/iASPP	Yes (prog-nostic)	Yes	Yes	(45–54, 56, 58–60, 62, 64–67)
		Apoptosis	Wnt/β-catenin signaling pathway miR140/iASPP				
		Autophagy	mTOR/ULK1 pathway				
		Invasion and metastasis	miR-342/Wnt5a/β-catenin (overall, it can modulate the Wnt/β-catenin signal pathway) miR-181d/β-catenin miR-152 miR-675/Cadherin13 miR140/iASPP				
		EMT	miR-130a-3p/SOX4 (regulating expression of N-cadherin & vimentin) Wnt/β-catenin signal pathway				
		Stemness	Expression of CD133, NANOG, Oct4, and SOX2				
		Angiogenesis	miR-342/Wnt5a/β-catenin miR-29a/VASH2 miR138/HIF-1α and VEGF Nkd1 (Wnt pathway inhibitor)				
**HOTAIR**	↑	Cell cycle and proliferation	EZH2/PRC2 miR-15-b/p53 NLK/β-catenin signaling pathway miR-219/Cyclin D1 miR-218/PDE7A miR125a/mTOR pathway miR-126-5p/glutaminase Expression of PDCD4	Yes	Yes	Yes	(91, 93, 96, 97, 99–108, 113)
		Apoptosis	miR-219/Bax miR-15-b/p53 miR-218/PDE7A miR125a/mTOR pathway				
		Invasion and metastasis	NLK/β-catenin signaling pathway miR-126-5p/glutaminase miR-15-b/p53 miR125a/mTOR pathway miR-218/PDE7A Expression of MMP-7 and MMP-9 Expression of PDCD4				
		Angiogenesis	Expression of VEGF miR-126-5p/glutaminase				
		Blood-tumor barrier	miR-148b-3p/USF1 (expression of ZO-1, occluding, claudin-5)				
		Metabolism	miR-126-5p (glutamine metabolism)				
**PVT1**	↑	Cell cycle and proliferation	miR-128-1-5p/PTBP1 miR-128-3p/GREM1 (inhibiting BMP signaling) miR−200a miR-190a-5p and miR-488-3p/MEF2C/JAGGED1	NR	Yes	Yes	(116–124, 130)
		Apoptosis	expression of Bcl-2, Bax, and caspase3 miR-128-3p/GREM1 (inhibiting BMP signaling) miR-128-1-5p/PTBP1 miR-190a-5p and miR-488-3p/MEF2C/JAGGED1				
		Autophagy	miR-187/Atg7 and Beclin1				
		Invasion and metastasis	miR-128-3p/GREM1 (inhibiting BMP signaling) miR-128-1-5p/PTBP1 (regulating expression of MMP-2 and MMP-9) miR-424 miR-190a-5p and miR-488-3p/MEF2C/JAGGED1 UFPF1				
		Angiogenesis	miR-26b/CTGF/ANGPT2 miR-187/Atg7 and Beclin1				
**UCA1**	↑	Cell cycle and proliferation	Wnt/β-catenin signal pathway miR182/iASPP miR-122 miR-135a/HOXD9	NR	Yes	Yes	(133, 135–141)
		Apoptosis	miR193a/CDK6 (blocking PI3K/AKT, MAPK, and Notch pathways) miR182/iASPP				
		Invasion and metastasis	Wnt/β-catenin signal pathway miR182/iASPP miR193a/CDK6 (blocking PI3K/AKT, MAPK, and Notch pathways) miR-135a/HOXD9 miR-204-5p/ZEB1 miR-122				
		EMT	miR-135a/HOXD9 miR-1 and miR-203a/Slug miR-204-5p/ZEB1				
		Stemness	miR-1 and miR-203a/Slug				
		Metabolism	miR-182/PFKFB2 (regulating glycolysis)				
**NEAT1**	↑↓	Cell cycle and proliferation	G1/S cell cycle transition miR-107/CDK6 miR-107/CDK14 miR-139-5p/CDK6 miR-132/SOX2 Wnt/β-Catenin Pathway miR-92b/DKK3 (tumor suppressor) miR-185-5p/DNMT1/mTOR signaling miR-98-5p/BZW1 miR-449b-5p/c-Met let-7e/NRAS	Yes	Yes	Yes	(147–161)
		Apoptosis	miR-139-5p/CDK6 miR-107/CDK6 miR-152-3p/CCT6A miR-92b/DKK3 (tumor suppressor) miR-185-5p/DNMT1/mTOR signaling let-7g-5p/MAP3K1 let-7e/NRAS				
		Invasion and metastasis	miR-139-5p/CDK6 miR-107/CDK14 miR-132/SOX2 miR-152-3p/CCT6A Wnt/β-Catenin Pathway miR-185-5p/DNMT1/mTOR signaling miR-449b-5p/c-Met let-7g-5p/MAP3K1 let-7e/NRAS				
		EMT	miR-185-5p/DNMT1/mTOR signaling				
		Stemness	miR-107/CDK6				
		Blood-tumor barrier	miR-181d-5p/SOX5/ZO-1, occludin, and claudin-5				
**XIST**	↑	Cell cycle and proliferation	miR-133a/SOX4 miR-204-5p/Bcl-2 miR-137-Rac1 miR-429 miR-152 miR-448/ROCK1 miR-329-3p/CREB1	NR	Yes	Yes	(162–172)
		Apoptosis	miR-204-5p/Bcl-2 miR-137-Rac1 miR-126/IRS1/PI3K/Akt pathway miR-329-3p/CREB1 miR-152				
		Invasion and metastasis	miR-133a/SOX4 miR-204-5p/Bcl-2 miR-126/IRS1/PI3K/Akt pathway miR-448/ROCK1 miR-329-3p/CREB1 miR-152				
		EMT	miR-133a/SOX4				
		Stemness	miR-152/KLF4				
		Angiogenesis	miR-137/FOXC1/CXCR7 miR-429				
		Blood-tumor barrier	miR-137/FOXC1 and ZO-2/ZO-1 and occludin				
		metabolism	Glucose:/miR-126/IRS1/PI3K/Akt pathway				
**CRNDE**	↑	Cell cycle and proliferation	miR-136-5p/Bcl-2-Wnt/PI3K/AKT/mTOR miR-186/PAK7/cyclin D1	NR	Yes	Yes	(173–179)
		Apoptosis	Bcl2/Bax expression ratio miR-136-5p/Bcl-2-Wnt/PI3K/AKT/mTOR miR-186/XIAP-PAK7/caspas3-BAD				
		Invasion and metastasis	miR-384/PIWIL4/STAT3 (expression of downstream molecules: cyclin D1, VEGFA, SLUG, MMP-9, Bcl-2, and bcl-xL) miR-136-5p/Bcl-2-Wnt/PI3K/AKT/mTOR miR-186/PAK7/MARK2				
		Immunity	TLR3-NF-κB-Cytokine(induced inflammation)				
**TUG1**	↑↓	Cell cycle and proliferation	G0/G1 phase transition	Yes (prog-nostic)	NR	Yes	(180–185)
		Invasion and metastasis	miR-26a/PTEN (tumor suppressor) miR-6321/ATF2				
		Stemness	miR-145/polycomb-mediated histone H3K27 methylation leading to suppression of differentiation genes				
		angiogenesis	miR-299/VEGF miR-6321/proangiogenic (VEGF, SDF-1) or antiangiogenic factors (PAI-1)				
		Apoptosis	activation of caspase-3 and-9, with inhibited expression of Bcl-2 (tumor suppressor) miR-26a/PTEN (tumor suppressor) Cell cycle arrest at the G0/G1 as a result of its knockdown				
		Blood-tumor barrier	miR-144/HSF2, ZO-1, occludin, and claudin-5 (tumor suppressor)				
**FOXD2-AS1**	↑	Cell cycle and proliferation	Decreasing recruitment ability of EZH2 to P53 miR-31/CDK1 miR-185-5p/CCND2 miR-98-5p/CPEB4 miR-185-5p/CCND2 miR-185/AKT1 miR-185-5P/HMGA2 (modulating PI3K/Akt signaling) miR-506-5p/Cyclin E1, CDK2, p21	NR	Yes	Yes	(186–194)
		Apoptosis	miR-98-5p/CPEB4 miR-185/AKT1				
		Invasion and metastasis	miR-185-5P/HMGA2 (modulating PI3K/Akt signaling) miR-98-5p/CPEB4 miR-185-5p/CCND2 miR-506-5p/MMP7, MMP9 miR-185/AKT1				
		EMT	miR-98-5p/CPEB4 miR-185-5p/CCND2/N-cadherin, vimentin and E-cadherin miR-506-5p/N-cadherin, vimentin and E-cadherin				
		Stemness	miR-185-5p/CCND2/Oct4, SOX2, and Nanog				
**ANRIL**	↑	Cell cycle and proliferation	ANRIL/let-7b-5p/JAK2/STAT3 miR-203a (regulating the activity of caspase-3/8/9 and the AKT signaling pathway) miR-34a/Sirt1 (activating the PI3K/AKT and mTOR signaling pathways)	NR	NR	Yes	(195–197)
		Apoptosis	miR-34a/Sirt1 (activating the PI3K/AKT and mTOR signaling pathways)				
		Invasion and metastasis	ANRIL/let-7b-5p/JAK2/STAT3 miR-34a/Sirt1 (activating the PI3K/AKT and mTOR signaling pathways)				
**HOXA11-AS**	↑	Cell cycle and proliferation	cell cycle transition at G0/G1 phase miR-140-5p miR-130a-5p/HMGB2 miR-125a miR-214-3p/EZH2 miR-124-3p	NR	NR	Yes	(198–203)
		Apoptosis	miR-130a-5p/HMGB2 miR-140-5p miR-125a/caspase-3/8/9, Bax, Gab2, and Bcl-2 miR-124-3p				
		Invasion and metastasis	miR-130a-5p/HMGB2 miR-125a miR-214-3p/EZH2 miR-124-3p				
**TP73-AS1**	↑	Cell cycle and proliferation	miR-103a/GALNT7 miR-124/iASPP miR-142/HMGB1/RAGE	NR	Yes	Yes	(204–206)
		Apoptosis	miR-103a/GALNT7 miR-124/iASPP				
		Invasion and metastasis	miR-124/iASPP miR-142/HMGB1/RAGE				
**DANCR**	↑	Cell cycle and proliferation	cell cycle transition at the G1/S and G0/G1 miR-216a/PI3K/AKT signaling pathway and LGR5 expression miR-634/RAB1A miR-135a-5p/BMI1 modulating AXL/PI3K/Akt/NF-κB pathway Wnt/β-catenin signaling miR-33a-5p	NR	Yes	Yes	(207–210)
		Apoptosis	miR-33a-5p/Bax and Bcl2				
		Invasion and metastasis	miR-216a/PI3K/AKT signaling pathway and LGR5 expression miR-135a-5p/BMI1 miR-33a-5p				
		EMT	miR-33a-5p (increased E-cadherin expression and decreased N-cadherin and Vimentin)				
		Angiogenesis	miR-216a/PI3K/AKT signaling pathway and LGR5 expression Wnt/β-catenin signaling				

Multiple studies reported increased expression and an oncogenic effect for *NEAT1* in glioma (147–160). *NEAT1* increased the activity of several signaling pathways with key roles in the cell cycle, including WNT/β-Catenin and mTOR signaling, leading to increased proliferation, invasion, and metastasis and decreased apoptosis. The interaction of *NEAT1* and EZH2, which mediates the trimethylation of H3K27 in their promoters, results in the activation of the WNT/β-Catenin pathway (156). Moreover, NEAT1 activated the mTOR signaling by acting as a ceRNA for *miR-185-5p* (158). *NEAT1* also altered the activity of some cell cycle regulators, including CDK6 and CDK14, *via* regulating the expression of miR-107 and *miR-139-5p*, which led to promotion of tumor proliferation, invasion and stemness, and inhibition of apoptosis (148, 149, 154). *NEAT1* also enhanced EMT *via* sponging *miR-185-5p* (158), and its knockdown resulted in increased BTB permeability by binding to *miR-181d-5p.* In contrast to these reports, Liu and colleagues found lower *NEAT1* expression in glioma tissue compared to the adjacent tissue. They found that *NEAT1* overexpression inhibited tumor proliferation and promoted apoptosis *via* regulating the *miR-92b*/DKK axis (161).

Moreover, several investigations found that *TUG1* had a higher expression in glioma and promoted tumor proliferation, invasion, stemness, and angiogenesis (180–183). TUG1 knockdown resulted in increased apoptosis and induced cell cycle arrest at G0/G1 (180). However, in Li et al.’s study, *TUG1* was downregulated in glioma (211). TUG1 acted as a tumor suppressor in few studies, with its downregulation inducing tumor proliferation and its overexpression resulting in increased apoptosis by triggering caspase-3 and caspase-9, inhibiting Bcl-2 (211), and upregulating PTEN (184). Additionally, *TUG1* knockdown increased BTB permeability through binding to *miR-144* (185).

*XIST*, another oncogenic lncRNA with increased expression in glioma, plays a major role in regulating the cell cycle leading to increased tumor proliferation and invasion and decreased apoptosis. Some of the underlying molecular mechanisms include regulating the expression of Bcl-2 *via* cross-talk with *miR-204-5p* (164), upregulating CREB1 *via* sponging *miR-329* (167), and regulating the insulin receptor substrate 1 (IRS1)/PI3K/Akt pathway *via* acting as a ceRNA for miR-126. Additionally, XIST also promoted EMT and tumor stemness *via* regulating the miR-133a/SOX4 and *miR-152*-Krüppel-like factor 4 (KLF4) axes, respectively. *XIST* also induced tumor proliferation and angiogenesis *via* inversely regulating *miR-429* (169) *and miR-137*. As a result of targeting *miR-137*, *XIST* knockdown also increased BTB permeability (168).

Unlike the oncogene lncRNAs, tumor suppressor lncRNAs are far less investigated. **Table 2** summarizes the most investigated tumor suppressor lncRNAs and the so far discovered intermediate molecular pathways, in addition to their clinical applications. *GAS5* (212–220), *CASC2* (221–224), *TUSC7* (225), and *MATN-AS1* (226, 227) are among these lncRNAs.

**Table 2:** The most investigated tumor suppressor lncRNAs and the so far discovered intermediate molecular pathways, in addition to their clinical applications.

lncRNA	Expression	Mechanism involved	Intermediate targets or signaling pathways	Clinical applications			References
				Circulatory biomarker	Treatment response	Prognostic
**MEG3**	↓	Cell cycle and proliferation	Modulating p53 expression and signaling WNT/β-catenin signaling pathway miR-19a/PTEN miR-377/PTEN miR-377/MTSS1 SMARCB/miR-6088 miR-21-3p Sirt7 (involved in the PI3K/AKT/mTOR signaling pathway)	NR	Yes	Yes	(71–73, 75, 77–81, 86)
		Apoptosis	Modulating p53 expression and signaling Expression of caspase 8/3 and TP53 Expression of Bax, cleaved caspase-3/-9, and Bcl-2				
		Autophagy	Regulating expression of Beclin-1, LC3, and p62				
		Invasion and metastasis	miR-19a/PTEN miR-377/PTEN miR-377/MTSS1 SMARCB/miR-6088 miR-21-3p				
		EMT	miR-377/PTEN Expression of N-cadherin, vimentin, Snail-1, and β- catenin, ZEB1/2				
**GAS5**	↓	Cell cycle and proliferation	miR-196a-5p/FOXO1/PID1 miR-18a-5p expression of GSTM3 miR-222/bmf/Bax, and Bcl-2 EZH2/PRC2/miR-424/AKT3 (cyclinD1, c-Myc, Bax, and Bcl-2)	Yes	Yes	Yes	(212–220)
		Autophagy	Regulating mTOR activation				
		Apoptosis	miR-196a-5p/FOXO1/PID1 expression of GSTM3 miR-10b/Sirtuin 1 miR-222/bmf/Bax, and Bcl-2 EZH2/PRC2/miR-424/AKT3 (cyclinD1, c-Myc, Bax, and Bcl-2)				
		Invasion and metastasis	miR-196a-5p/FOXO1/MIIP miR-18a-5p expression of GSTM3 miR-10b/Sirtuin 1/PTEN-PI3K-AKT and MEK-ERK cascades miR-222/bmf/Bax, and Bcl-2 miR-222/Plexin C1/cofilin EZH2/PRC2/miR-424/AKT3 (cyclinD1, c-Myc, Bax, and Bcl-2)				
		EMT	miR-106b/PTEN				
**CASC2**	↓	Cell cycle and proliferation	miR-18a miR-21 miR-181a/PTEN Pathway Wnt/β-catenin signaling pathway	NR	YES	Yes	(221–224)
		Apoptosis	miR-18a miR-21				
		Autophagy	miR-193a-5p/mTOR				
		Invasion and metastasis	miR-18a miR-21 Wnt/β-catenin signaling pathway				
		EMT	miR-18a				
**TUSC7**	↓	Cell cycle and proliferation	miR-23b	Yes (prog-nostic)	Yes	Yes	(225)
		Apoptosis	miR-23b				
		Invasion and metastasis	miR-23b				
MATN1-AS1*	↑↓	Cell cycle and proliferation	RELA (also known as p65) (involved in MAPK signaling pathway) miR‐200b-c-429/CHD1 (Tumor inducer)	NR	Yes	NR	(226, 227)
		Apoptosis	RELA (involved in MAPK signaling pathway) miR‐200b-c-429/CHD1 (Tumor inducer)				
		Invasion and metastasis	RELA (involved in MAPK signaling pathway)				

Second to *MEG3*, the anti-tumoral effects of *GAS5* are well investigated in glioma. *GAS5 has a* major role in cell cycle regulation with several mechanisms. For instance, *GAS5* regulated the expression of tumor suppressors Bcl-2-modifying factor (Bmf) and Plexin C1 *via* targeting *miR-222*, which led to inhibition of tumor progression (215). *GAS5* also inhibited tumor inducer *miR-196-5p*, which led to suppressed tumor growth by positive regulation of tumor suppressors forkhead box protein O1 (FOXO1) and phosphotyrosine interaction domain containing 1 (PID1) (213). Direct interaction of *GAS5* and EZH2, in addition to the promotion of *miR-424* expression, are among other putative mechanisms for the tumor-suppressing effect of *GAS5*. *miR-424* inhibited AKT3 and regulated the expression of cyclin D1, c-Myc, Bax, and Bcl-2 (214). Furthermore, *GAS5* was found to inhibit excessive autophagy in glioma (216).

Moreover, the function of *MATN1-AS1* is controversial in glioma. Han et al. found lower expression of *MATN1-AS1* in glioblastoma tissue compared to the adjacent tissue, acting as a tumor suppressor (226). Its overexpression inhibited proliferation and promoted apoptosis (226). On the other hand, Zhu and colleagues found higher *MATN1-AS1* expression in glioma tissue and cell lines (227). They reported oncogene effects for this lncRNA, with its silencing inhibiting proliferation and promoting apoptosis./

**Figure 2:**
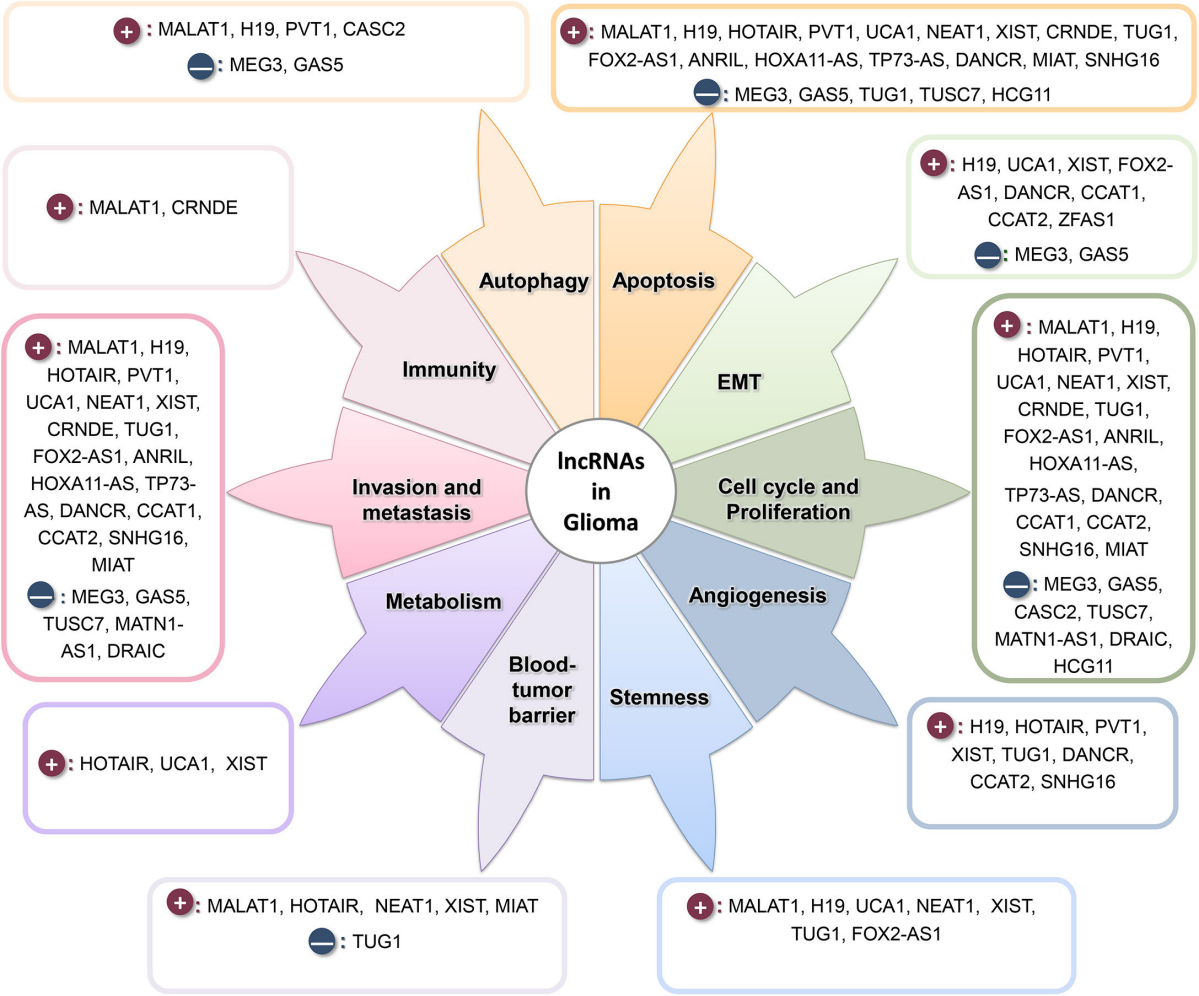
lncRNAs play a prominent role in various stages of tumor formation and progression. Dark-red circles show the oncogene lncRNAs, and navy circles show the tumor suppressor lncRNAs.

As depicted in **Figure 2**, lncRNAs are involved in almost all stages of tumorigenesis (228). In addition to the lncRNAs described in **Tables 1** and **2**, we have included other oncogene lncRNAs, which are upregulated in glioma, such as *CCAT1* (229), *CCAT2* (230), *SNHG16* (231, 232), *MIAT* (233, 234), *DRAIC* (235), and *HCG11* (236) in this Figure.

#### Clinical Applications

**Circulatory biomarker**: Higher circulatory levels of *NEAT1* (151), *LINK-A* (237), and *AWPPH* (238), in addition to lower serum *GASL1* levels (239), have shown considerable diagnostic value for glioma.

**Figure 3:**
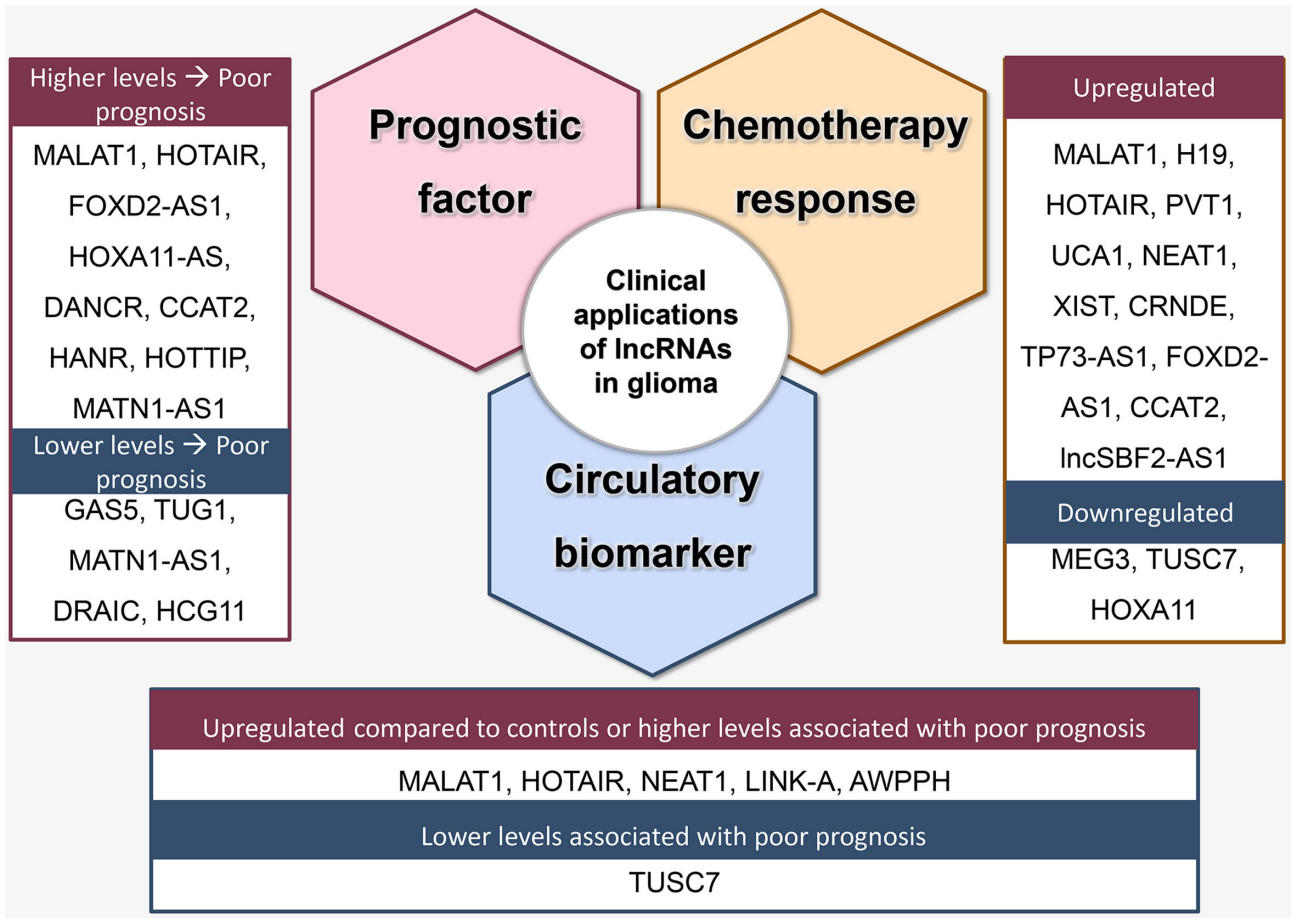
Clinical applications of lncRNA as prognostic factors, predictors of treatment response, and circulatory biomarkers.

In addition to diagnosis, circulatory levels of lncRNAs can also aid in determining prognosis. Lower circulatory levels of *GAS5* (40) and *TUSC7* (240) and higher circulatory levels of *AWPPH* (238) were associated with poor prognosis. Moreover, levels of circulating lncRNAs may also illuminate treatment response. For instance, higher levels of *lncSBF2-AS1* in serum exosomes were associated with poor TMZ-response (241). The most investigated lncRNAs with either diagnostic or prognostic value for their circulatory levels are described in **Figure 3**.

**Prognostic value**: Higher expression of *NEAT1* (147), *XIST* (164), *CRNDE* (173, 179), *FOXD2-AS1* (186, 189, 192, 194), *ANRIL* (196), *HOXA11-AS* (198–200, 203), *TP73-AS1* (204, 242), and *DANCR* (208, 210, 243), in addition to lower expression of *GAS5* (220), *CASC2* (222, 244), *TUSC7* (240) correlated with a more advanced stage of disease or poor survival (**Figure 3**). Notably, controversial findings were reported for some lncRNAs. For *MATN1-AS1*, while Zhu et al. reported a positive association between its upregulation and tumor advancement and reduced overall survival (227), Han et al. reported that its downregulation was a poor outcome predictor (226). Moreover, while several studies reported an oncogenic effect for *TUG1*, Wang et al. found that *TUG1* expression negatively correlated with tumor grade (245). In contrast to *HOXA11-AS*, for which several studies reported a positive correlation with poor prognosis, low expression of HOXA11, which is within the same family, was associated with poor outcome in glioblastoma (246). Additionally, higher levels of *CCAT2* (247), *HOTTIP*, *HANR*, and lower levels of *DRAIC* and *HCG11* were also associated with poor prognosis (248).

In addition to the expression level, SNPs of some lncRNAs may also provide prognostic information. For example, specific *ANRIL* SNPs were related to the susceptibility of glioma and patients’ overall survival (249, 250).

**Determining treatment response**: lncRNAs modulate treatment response by affecting sensitivity to chemotherapy drugs, mainly TMZ or cisplatin, or altering radiosensitivity. Higher *NEAT1* (151, 160), *XIST* (172), *FOXD2-AS1* (189, 192), *TP73-AS1* (251), *CCAT2* (252), and *lncSBF2-AS1* (241) were associated with TMZ-resistance. These effects are mediated *via* several mechanisms. For instance, *NEAT1* promoted glioma stem cell formation, which is critical for chemoresistance, *via* activating the Wnt/β-catenin pathway (160). In another example, *FOXD2-AS1* reduced methylation and increased activity of O^6^-methylguanine-DNA methyltransferase (MGMT), which is a treatment response predictor in glioma (192). Furthermore, cisplatin resistance was associated with higher levels of *DANCR* (253), *CRNDE* (178), *CCAT2* (252), and lower levels of *GAS5* (216). Additionally, overexpression of *XIST* reduced radiosensitivity (167), while high expression of *DRAIC* was associated with a better prognosis of radiotherapy in low-grade glioma (254).

***In-vivo* therapeutic applications**: For many oncogenic lncRNAs, including *XIST* (164, 169, 171), *NEAT1* (150, 158), *CRNDE* (173, 175, 176), *TUG1* (182, 183), *FOXD2-AS1* (190), *HOXA11-AS* (199, 201), and *TP73-AS1* (204, 208), and tumor suppressor lncRNAs, including *GAS5* (214, 215), *CASC2* (221), *MATN1-AS1* (226), animal studies, which are more advanced stages of investigating roles of lncRNAs (255), validated their effect on glioma. Furthermore, lncRNAs can mediate the effects of anti-cancer drugs. For instance, the anti-tumoral effect of sevoflurane was mediated through regulating the *ANRIL*/let-7b-5p axis (195).

## Discussion

Given the mounting and emerging evidence on the roles of lncRNAs in different cancers, including glioma, this review provided a comprehensive summary of the mechanisms of action and clinical relevance of the most investigated lncRNAs in glioma. A profound understanding of the underlying molecular pathways involved in the function of lncRNAs is required to develop novel therapeutic targets. As described earlier, several lncRNA/miRNA/mRNA axes have been proposed to mediate the oncogenic or tumor suppressor effects of lncRNAs. In addition to well-known roles and associations with prognosis and treatment response in various cancers, lncRNAs can provide clinical clues in several non-neoplastic diseases, such as neurodegenerative disease (256) and cardiovascular disease (257, 258). The disrupted pattern of lncRNAs in a wide spectrum of cancers raises the question of whether the roles and associations identified in a particular type of malignancy can be expanded to the other cancer types. A recent study found that while some lncRNAs are consistently associated with better or poorer prognosis across different cancer types, some other lncRNAs show different associations in various cancers (259).

LncRNAs hold promise for developing novel biomarkers and therapeutic targets. To the best of our knowledge, prostate cancer antigen 3 (*PCA3*) is the only lncRNA approved as a diagnostic biomarker for prostate cancer in clinical practice (260). All in all, given the high specificity of tissue/serum lncRNAs in glioma, they may be excellent candidates for novel biomarkers. LncRNAs secreted as exosomes in body fluids, especially serum, can provide novel non-invasive assessment tools (255). Two main approaches are commonly utilized to modulate the expression of lncRNAs, namely antisense oligonucleotides (ASOs) and duplex RNAs, such as siRNA. ASOs may be preferred for lncRNAs functioning in the nucleus, while siRNA may be selected for lncRNAs functioning in the cytoplasm (261). While oligonucleotide therapeutics provide an opportunity to target any gene of choice, there are several obstacles in their clinical use. As a consequence of their chemical structure, they are susceptible to rapid enzymatic and nonenzymatic degradation. Moreover, their relatively large size hinders their penetration through the blood-brain barrier and cellular uptake. Therefore, novel delivery systems, such as chemical engineering and nanoparticles, are needed to overcome these challenges (262, 263).

Despite considerable attention drawn to lncRNAs in cancer and the growing evidence, there are several gaps in the literature. The findings of some studies are not sufficiently reliable due to small sample sizes. Moreover, the majority of investigations are *in-vitro* assessments highlighting the need for further validation by nude animal models and clinical trials. However, the low conserveness of lncRNAs among different species may hinder using animal models since the function of a certain lncRNA can be different between humans and animal models. As a result, engineered models may be required (261).

Our study shed light on several directions for future studies. In addition to the need for increased *in-vivo* investigations and studies with larger sample sizes, it is not elucidated whether disruption in expression of lncRNAs is a culprit or consequence of the malignancy. Future studies need to address this question, particularly by investigating upstream regulators of the expression of lncRNAs. Moreover, the development of specific panels of lncRNAs for diagnostic or prognostic applications can lead to increased specificity and sensitivity. Several studies have already taken this approach and sought to find specific signatures of lncRNAs for glioma (264–266). Additionally, more studies are required on the therapeutic effects of combining the lncRNA-targeted therapies and conventional chemotherapy. For instance, a better outcome was achieved after administrating si-*MALAT1* in addition to TMZ *in vitro* and in animal models (16). Additionally, detecting the disease-associated lncRNAs and their regulatory pathways can also lead to finding novel putative drugs (267). Lastly, the advancement of computational technologies and bioinformatics have provided novel opportunities for identifying new lncRNAs and their potential molecular mechanism (268). Applying artificial intelligence technology, including machine-learning and deep-learning models, can also aid in the identification of novel lncRNAs associated with a specific disease mainly *via* classification models (269).

To conclude, regulating diverse cellular signaling pathways and the expression of various proteins involved in different stages of tumor formation, proliferation, and invasion, lncRNAs are potential candidates for developing novel diagnostic, prognostic, and therapeutic approaches. The so far discovered associations between their expression in the tissue or circulatory exosomes with treatment response and prognosis boost hopes for their potential use in clinical practice. However, despite substantial advances, the role of lncRNAs in glioma remains fairly unknown. It is not well known whether the disruption in the expression of lncRNAs has a causal effect on glioma or is a consequence of the malignant process. Moreover, some controversial reports hinder drawing a concrete conclusion. More investigations with larger sample sizes and increased focus on *in-vivo* models are required to expand our understanding of the potential roles and application of lncRNAs in glioma.

## Author Contributions 

SM: Conceptualization, Investigation, Writing - Original Draft, and Visualization. NR: Conceptualization, Writing - Review & Editing, Supervision. All authors contributed to the article and approved the submitted version.

## Conflict of Interest

The authors declare that the research was conducted in the absence of any commercial or financial relationships that could be construed as a potential conflict of interest.

## Glossary

**Table:**

ABCG2	ATP-binding cassette subfamily G member 2
ANGP2	angiopoietin 2
ANRIL	antisense RNA in the INK4 locus
ASO	antisense oligonucleotide
ATF-2	activating transcription factor-2
AWPPH	associated with poor prognosis of hepatocellular carcinoma
BMP	bone morphogenetic protein
BTB	blood tumor barrier
CASC2	cancer susceptibility candidate 2
CCAT	colon cancer-associated transcript
CD	cluster of differentiation
ceRNA	competing endogenous RNA
CLOCK	circadian locomotor output cycles kaput
CRNDE	colorectal neoplasia differentially expressed
CTGF	connective tissue growth factor
DANCR	differentiation antagonizing non-protein coding RNA
DNA	deoxyribonucleic acid
DNMT1	DNA (cytosine-5)-methyltransferase 1
DRAIC	downregulated RNA in cancer
EMT	epithelial-mesenchymal transition
ERK	extracellular signal-regulated kinases
EZH2	enhancer of zeste homolog 2
FBXW7	F-box and WD repeat domain containing 7
FOXO1	forkhead box protein O1
GAS5	growth arrest-specific transcript 5
GASL1	growth-arrest-associated lncRNA 1
GOLM1	Golgi membrane protein 1
GREM1	Gremlin 1
GSTM3	Glutathione S-Transferase Mu 3
GTL2	gene-trap locus 2
HCG11	human leukocyte antigen complex group 11
HIF	hypoxia-inducible factor
HMGB1	high mobility group box 1 protein
HOTAIR	HOX transcript antisense intergenic RNA
HOXA11-AS	HOXA11 antisense RNA
HSP	heat shock protein
iASPP	inhibitor of apoptosis-stimulating protein of p53
IDH	isocitrate dehydrogenase
IRS1	insulin receptor substrate 1
KLF4	Krüppel-like factor 4
KPS	Karnofsky performance score
LGR5	Leucine-rich repeat-containing G-protein coupled receptor 5
LINK-A	long intergenic non−coding RNA for kinase activation
lncRNA	long non-coding RNA
MALAT1	metastasis-associated lung adenocarcinoma transcript 1
MAPK	mitogen-activated protein kinase
MEF2C	myocyte-specific enhancer factor 2C
MEF2C	myocyte enhancer factor 2C
MEG3	maternally expressed gene 3
MGMT	O6-methylguanine-DNA methyltransferase
MIAT	myocardial infarction−associated transcript
MIIP	migration and invasion inhibitory protein
miRNA	microRNA
MMP	matrix metalloproteinase
mRNA	messenger RNA
MRP1	multi-drug resistance (MDR)-associated protein 1
mTOR	mammalian target of rapamycin
MTSS1	metastasis suppressor 1
NEAT1	nuclear paraspeckle assembly transcript 1
NFYA	nuclear factor YA
NLK	Nemo-like kinase
Oct4	octamer‐binding transcription factor 4
PCA3	prostate cancer antigen 3
PDCD4	programmed cell death 4
PFKFB2	6-phosphofructo-2-kinase/fructose-2,6-bisphosphatase 2
PI3K	phosphoinositide 3-kinase
PID1	phosphotyrosine interaction domain containing 1
PRC2	polycomb repressive complex 2
PTBP1	polypyrimidine tract-binding protein 1
PTEN	phosphatase and TENsin homolog
PVT1	plasmacytoma variant translocation 1
RNA	ribonucleic acid
SNHG	small nuclear RNA host gene
SOX	sex determining region Y‐box
STMN1	Stathmin 1
TMZ	temozolomide
TNM	tumor
node	metastasis
TP73-AS1	TP73 antisense RNA 1
TUG1	Taurine upregulated gene 1
TUSC7	tumor suppressor candidate 7
UCA1	Urothelial carcinoma associated 1
ULK1	Unc-51 like autophagy activating kinase 1
UPF1	up-frameshift protein1
USF1	upstream stimulatory factor 1
VASH2	vasohibin-2
VDR	vitamin D receptor
VEGF	vascular endothelial growth factor
WHO	world health organization
XIST	X-inactive specific transcript
ZEB2	zinc finger E−box binding homeobox 2
ZHX1	zinc-fingers and homeoboxes 1
